# 

**Published:** 1862-06

**Authors:** 


### CONTENTS.

### ORIGINAL CONTRIBUTIONS.

ART. XVI. RETAINED PLACENTA. By F. R. PAYNE, M.D............ 331

ART. XVII. The Influence of Marriages of Consanguinity on the Offspring. By E. A. NEYMAN, Student of Med. Depart. of Lind University........ 338

ART. XVIII. Letter from E. ANDREWS, M.D. ect........ 342

### THE CLINIQUE.

Mercy Hospital, Service Prof. J. H. HOLLISTER, Attending Surgeon. Popliteal Aneurism........ 346

### PROCEEDINGS OF SOCIETIES.

Chicago Medical Society—Regular Meeting, June 6, 1862. A very rare Gun-Shot Wound. Reported by CHARLES G. SMITH, M.D.... 354

### SELECTIONS.

Cases of Vagismus, with the method of treatment. By J. MARION SIMS, M.D........ 355

Cantharides as a Therapeutical Agent, Internally Administered in Large Doses. Its Mode of Operation. By Alex. McBride, M.D., Surgeon O.V., U.S.A........ 364

Paracentesis in Hydrothorax........ 372

Marriages of Consanguinity........ 374

Arsenic and Sesquicarbonate of Ammonia in Ague........ 375

### BOOK NOTICES.

A Manual of Medical Diagnosis: Being an Analysis of the Signs and Symptoms of Disease. By A. W. Barclay, M.D., etc........ 376

Researches and Observations on Pelvic Hæmatocele. By J. Bryne, M.D., ect........ 376

Hints and Observations on Military Hygiene, with the best means of Treating the Medical and Surgical Diseases of the Army. By Laurence Trumball, M.D., etc........ 380

### EDITORIAL.

Sanitary Condition of Camp Douglas. By J. H. Hollister, M.D........ 381

Changes in the Medical Department of Lind University........ 389

Changes at Camp Douglas........ 389

Army Medical Officers not Belligerents........ 390

Discussion of Dr. J. M. Sim's paper on Vaginismus........ 390

Taking Cold, etc........ 392

Army Sanitary Inspectors........ 393

Surgeons and Nurses........ 393

Recent Acts of Congress........ 393

Meeting of Pensylvania State Medical Society........ 393

